# Teriparatide as a nonoperative treatment for tibial and femoral fracture nonunion: A case report

**DOI:** 10.1097/MD.0000000000006571

**Published:** 2017-04-21

**Authors:** Li Xiaofeng, Xu Daxia, Chen Yunzhen

**Affiliations:** Department of Orthopedics, Qilu Hospital of Shandong University, Ji’nan, Shandong Province, China.

**Keywords:** bone metabolic markers, fracture healing, nonunion, teriparatide

## Abstract

**Rationale:**

Fracture nonunion is a great challenge for orthopedic surgeons. Many surgical interventions are associated with significant pain and heavy economic burden. Therefore, our aim was to evaluate the outcomes of a new nonoperative treatment for fracture nonunion.

**Patient concerns:**

A 44-year-old man suffered closed fractures of the right tibia and left femur. Eleven months after surgery, there was no radiographic healing between fracture fragments.

**Diagnoses:**

Fracture nonunion of the right tibia and left femur.

**Interventions:**

The patient received systemic treatment with teriparatide (recombinant human Parathyroid Hormone 1–34) 20 μg/d for 8 months, with further observation at 4 months after discontinuation. During treatment, bone metabolic markers were measured to evaluate metabolic activity of osteoblasts and osteoclasts. The Ethics Committee of Qilu Hospital of Shandong University approved this study.

**Outcomes:**

Satisfactory healing of fracture nonunion was obtained without further intervention.

**Lessons:**

Anabolic treatment with teriparatide showed a positive effect on healing of fracture nonunion. Evaluation of bone metabolic markers during treatment is necessary to observe the curative effect. In view of the positive effect of teriparatide on healing of fracture nonunion in numerous animal models and clinical studies, it may be a promising alternative treatment for fracture nonunion in patients who are not suitable for surgical intervention.

## 1 Introduction

Teriparatide was licensed for treatment of postmenopausal osteoporosis by the US Food and Drug Administration in 2002.^[1]^ In recent years, the efficacy of teriparatide in promoting fracture healing has been reported in numerous animal models ^[2,3]^ and clinical studies.^[4–6]^ Fracture nonunion remains a great challenge for orthopedic surgeons. To date, many surgical interventions are associated with significant pain and heavy economic burden. Therefore, based on the positive effect of teriparatide on healing of fracture delayed union or nonunion in previous studies, we present a case of tibial and femoral fracture nonunion treated with teriparatide, which obtained successful healing.

## 2 Case report

**Figure 1:**
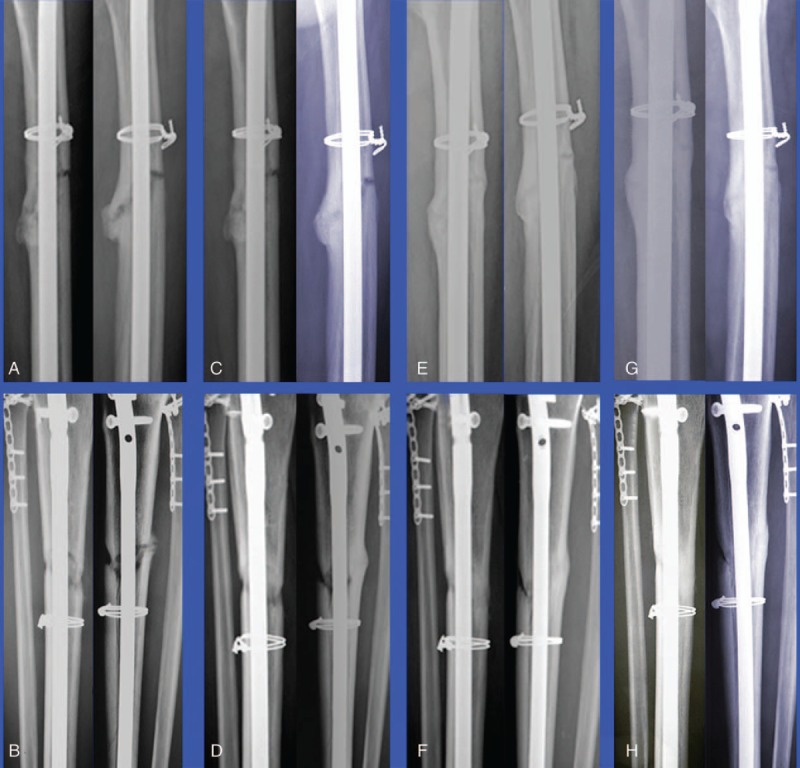
Serial anteroposterior and lateral plain radiographs of left tibial and right femoral fracture nonunion during treatment. (A and B) Nonunion before treatment. (C and D) Nonunion after a 4-month course of teriparatide. (E and F) Nonunion after an 8-month course of teriparatide. (G and H) Healing of nonunion at 4 months after discontinuation of teriparatide.

A 44-year-old man suffered closed fractures of the right tibia and left femur in a motor vehicle accident in January 2015. He underwent retrograde intramedullary nailing at another hospital. He had no postoperative infection. Medical history taking revealed no chronic diseases, hypertension, diabetes mellitus, smoking, alcohol abuse, or regular medication. Until 10 months postoperatively, there was no radiographic healing of the right tibia, and the patient experienced persistent pain during weight bearing. Radiographs obtained 11 months postoperatively revealed a persistent fracture gap, sclerosis of the fracture margin, and no evidence of bone bridging at the fracture site (Fig. 1A and B). Therefore, a diagnosis of nonunion was made. There was no clinical sign of infection based on erythrocyte sedimentation rate, C-reactive protein level, and leukocyte count. Other laboratory tests, including parathyroid hormone (PTH), serum alkaline phosphatase, phosphorus, calcium, and 1,25-(OH)_2_D_3_, were within normal range. The patient refused to undergo another operation; instead, off-label use of teriparatide was accepted (20 μg ih qd). During the 8 months of treatment, levels of PTH, C-terminal telopeptide of type I collagen (CTX), and N-terminal propeptide of type I collagen (P1NP) were measured at baseline, 1 month, 4 months and 8 months, and at 4 months after discontinuation (Table 1). P1NP and CTX levels were significantly elevated at baseline and maintained a steady state during the treatment period. No adverse effects were observed during or after treatment. After 4 months of treatment, radiographs showed a decrease of the fracture gap and bone bridging between fracture fragments (Fig. 1C and D). After 8 months of treatment, radiographs revealed continuous improvement of the fracture gap (Fig. 1E and F). Then, teriparatide was discontinued. Four months after discontinuation, radiographs showed complete fracture union (Fig. 1G and H). The patient's pain disappeared, and he returned to normal activity.

**Table 1:**
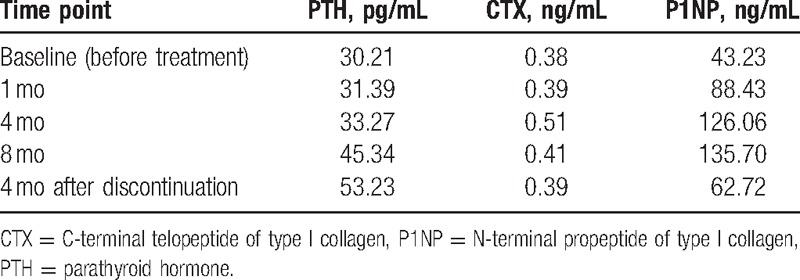
Changes in bone metabolic markers.

## 3 Discussion

Teriparatide, as a bone-forming agent, has been used to treat osteoporosis and has become a promising therapeutic agent to improve healing of fracture nonunion. The primary physiologic mechanism of teriparatide involves activation of osteoblasts; intermittently administered teriparatide can shift the bone resorption of osteoclasts to the activation of osteoblasts.^[7–9]^ Teriparatide plays an important role in the coupling of osteolysis and osteogenesis and is an indispensable regulator in remodeling. Teriparatide has been demonstrated to improve the microarchitecture of bone, increase bone mineral density, and reduce the risk of vertebral and nonvertebral fractures.^[10]^ Consistent with these pharmacokinetic characteristics, a rapid increase in biomarkers of bone formation (P1NP) and a gradual increase in biomarkers of bone resorption (CTX) have been observed, demonstrating activation of the metabolic activity of osteoblasts and osteoclasts, referred to as the bone anabolic window.^[11]^ Bone turnover markers are widely used to evaluate metabolic disease, such as Paget disease and osteoporosis, but are used less often to evaluate healing of fracture nounion.^[12]^ However, measurement of the specific molecular markers of osteoblasts and osteoclasts could improve the evaluation of fracture healing and detect the risk of nonunion.^[13]^ P1NP may be a promising marker of nonunion, with 1 previous study showing that it decreased to normal levels during union.^[14]^ In that same study, the changing rate of bone collagen synthesis was closely related to the changing rate of bone formation during fracture healing.

Prominent improvements in callus mineralization and volume, bone mineral content, and rate and strength of successful healing between fracture fragments have been demonstrated in both delayed union and nonunion models.^[15]^ The important role of teriparatide was confirmed in a study by Ren et al,^[16]^ who showed a lack of endogenous PTH (1–84) restrained fracture healing, whereas, if exogenous recombinant human Parathyroid Hormone (1–34) was combined with endogenous PTH (1–84), endochondral bone formation, callus remodeling, and mechanical bone strength were improved to promote fracture healing. Case reports ^[17,18]^ also have shown the advantages of teriparatide in cases of fracture nonunion that have innate difficulty in achieving healing. These results imply that teriparatide works effectively when the fracture gap is union, either surgical fixation or nonsurgical.

Today, correlational studies are limited by the deficiency of systematic studies. In view of the limited valid evidence regarding use of teriparatide in cases of chronic fracture nonunion, we depended considerably on retrospective case series and case reports. Nevertheless, the anecdotal evidence of the positive effect of teriparatide on healing of fracture nonunion offers guidance for clinical decision making. Better understanding of this effect may be expounded in future prospective experiments. However, based on our case, teriparatide is a promising anabolic therapy to improve healing of fracture nonunion in patients who are not suitable for surgical intervention.

## Acknowledgments

The authors would like to thank the patient and his family for allowing us to use the medical documentation and information that led to the present article.
